# A simple and interpretable method to predict head and neck offline adaptation needs based on anatomical changes

**DOI:** 10.1002/mp.70732

**Published:** 2026-10-22

**Authors:** Tim Flühmann, Gian Guyer, Véronique Vallet, Stina Svensson, Anna Lundin, Raphaël Moeckli, David Patin

**Affiliations:** ^1^ Institute of Radiation Physics Lausanne University Hospital and University of Lausanne Lausanne Switzerland; ^2^ Department of Physics ETH Zürich Zürich Switzerland; ^3^ RaySearchLabs Stockholm Sweden; ^4^ Present address: Department of Diabetes, Endocrinology, Nutritional Medicine and Metabolism, Inselspital University of Bern Bern Switzerland

**Keywords:** offline ART, replanning prediction

## Abstract

**Background:**

Modern radiotherapy techniques do not automatically account for anatomical changes during treatment, which can lead to reduced target coverage, hotspots, or increased dose to healthy tissues. Adaptive Radiotherapy (ART) addresses this by updating the treatment plan based on the patient's current anatomy. In routine clinical practice, the decision to replan is often triggered by a suboptimal fit of the immobilization mask, identified by the radiation therapy technologist. Because this process is operator dependent and lacks methodological rigor, a quantitative decision‐support method could substantially improve the replanning decision. Daily image‐guided radiotherapy (IGRT) images provide valuable clinical information but often lack sufficient quality for accurate target contouring or reliable dose calculation to support straightforward clinical decision‐making. This limitation motivates the need for a simpler, more practical, and scalable alternative.

**Purpose:**

This study develops an automated workflow to predict the need for replanning at every treatment fraction in head and neck (H&N) cancer patients using robust tools, thus requiring minimal manual validation while aiding the radio‐oncologists in their decision‐making.

**Methods:**

A retrospective dataset of 21 H&N cancer patients treated on a Tomotherapy Radixact system (Accuray, USA) with kVCT daily imaging was analyzed, of whom 14 underwent replanning during treatment. For each patient, the planning CT (pCT) and daily images were processed in RayStation treatment planning system (RaySearch Laboratories AB, Sweden) via scripts. Rigid registrations were used to align daily images to the pCT, and a patient‐specific region of interest (ROI) was defined to consistently capture the same anatomical region across all treatment fractions. ROI volumes were intersected with the external contour and converted to relative volumetric changes with respect to the pCT. By grouping patients according to whether a replanning occurred during treatment, differences in mean volumetric change between replanned and non‐replanned patients were assessed at each treatment fraction. To automatically detect clinically relevant anatomical changes, a simple, interpretable model based on three volumetric features (current volume loss, short‐term trend, and long‐term trend) was evaluated using cross‐validation. Thresholds defining the trigger condition were optimized using a tailored loss function that prioritizes early detection and penalizes false negatives more strongly than false positives. Model variants using different feature subsets and hyperparameters were compared, and the final configuration was selected prioritizing recall, followed by specificity and early flagging.

**Results:**

Significant differences in volumetric change between replanned and non‐replanned patients emerged at fraction 6 and remained significant for most subsequent fractions. The smallest adjusted p‐values occurred around fractions 22–25. Using leave‐one‐out cross‐validation, the best‐performing model flagged patients to be replanned with 93% recall, doing so on average 8.4 fractions before clinical intervention.

**Conclusions:**

Consistent differences in local tissue volume loss between replanned and non‐replanned patients enabled the development of a simple automated workflow that triggers replanning for offline ART when learned volumetric thresholds are exceeded. This approach provides an objective complement to physician judgement and integrates seamlessly into existing IGRT workflows.

## 1 INTRODUCTION

In view of increasing life expectancy, population growth, and rising exposure to risk factors, cancer diagnoses are predicted to rise by 77% between 2022 and 2050.^1^ With these rates, treatment costs and the need for better cancer treatment are also growing, which requires a well‑structured allocation of resources. To support the treating professionals, recent establishments in Artificial Intelligence and computerized decision‑making can be exploited, potentially saving both time and costs while simultaneously improving the quality of treatment.^2^

As a major advancement in radiotherapy, which is part of the treatment for more than 50% of all cancer patients,^3^ Image‑Guided Radiotherapy (IGRT) ensures consistent patient positioning and ultimately allows for minimal safety‑margins.^4^ Furthermore, the reconstructed 3D images from IGRT can shed light on anatomical changes that have occurred since the start of the treatment, helping the radio‐oncologists argue for the safe continuation with the original plan or the need for replanning.^5^

Adaptive Radiotherapy (ART) is based on adapting treatment plans during the course of radiotherapy treatment when needed. ART can be performed offline or online. While offline ART is typically implemented between treatment fractions, online ART is applied immediately before a fraction with the patient waiting on the treatment table. Advantages of offline ART include that the treatment does not require additional time on the machine, allowing more patients to be treated during a day. After a replanning is determined, the necessary modifications are made and integrated into a revised plan. In contrast, online ART is performed just before radiation while the patient is already in the treatment position. The advantage is that observed changes can be answered directly and the treatment plan adapted. However, this requires a very efficient and well‑integrated workflow,^6^ including a dedicated team consisting of radio‐oncologists, medical physicists and technicians making the right decisions and the work of contouring, planning and validation in a short amount of time.

Although ART was introduced over 20 years ago, no single ideal approach has definitively emerged as superior, and workflows may vary significantly across centers and tumor sites.^6^ Additionally, the replanning procedure is dependent on the available resources and can amount to high costs.^7^ Sometimes, retrospective analysis of patients who underwent replanning reveals that their treatment still met clinical goals, making the replanning unnecessary.^8^ To avoid this inefficiency and preserve resources, a reliable method should be implemented to support radio‐oncologists in their decision‑making.

Current decision‑making regarding the replanning of head and neck (H&N) cancer patients can be mainly based on mask fitting, weight change, or image‑based assessments.^5^, ^9^ Unlike unfitting masks and weight loss, image‑based methods are not as straightforward. In many cases, they include deformable image registrations (DIR) between daily positional images and the primary planning computed tomography (pCT) scan or perform new simulation scans in either regularly planned intervals or when gross anatomical changes are suspected.^8^, ^9^ Given that the need for replanning has been found to be correlated with weight loss,^9^, ^10^, ^11^, ^12^ some methods quantify anatomical changes in the H&N region through surrogate measurements such as neck diameter tracked over the course of treatment.^13^

In this study, we propose an automated approach to trigger offline ART for H&N cancer patients, based on volumetric changes assessed through daily imaging (see overview in Figure 1). The approach is interpretable by construction, relies on robust manipulations and needs minimal validation. The contributions are twofold: First, the hypothesis that replanning necessity can be predicted by evaluation of volumetric changes in the H&N region on daily images is tested by comparing cohorts with and without replanning. Second, a model based on features derived from the relative volumetric changes is constructed to predict the need for plan adaptation during treatment.

**FIGURE 1:**
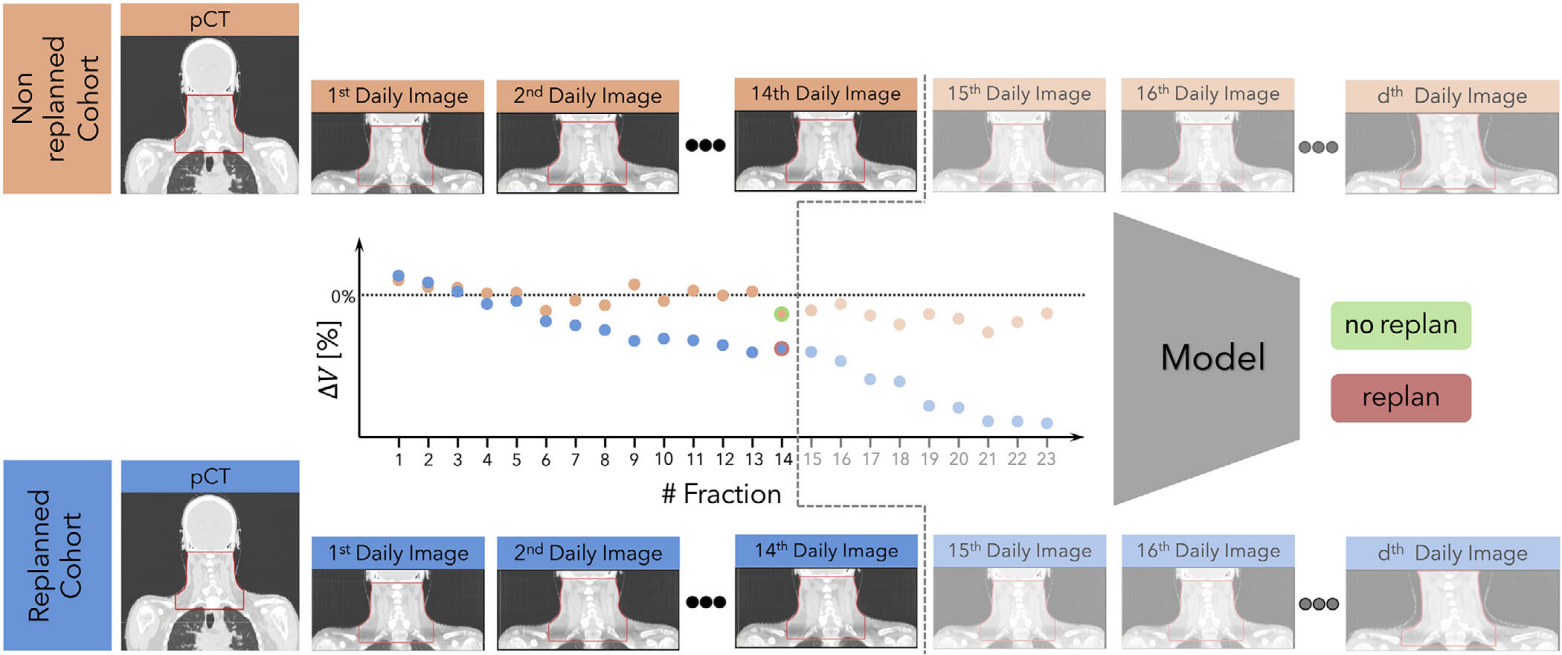
Offline adaptive radiotherapy for H&N cancer patients through local volumetric changes. 1. Patients are grouped according to whether replanning occurred, and daily volumetric changes are quantified by constructing consistent ROIs on all images and computing the relative difference to the planning CT images. Statistical tests identify fractions with significant differences between groups. 2. A simple predictive model is trained to sequentially evaluate all available fractions per patient and determine whether replanning should be triggered or whether continuation with the current treatment plan remains appropriate.

## 2 MATERIALS AND METHODS

A dataset was created comprising H&N cancer patients, some of whom underwent replanning. For each patient, the planning CT (pCT) as well as kVCT daily images were available. To assess volumetric changes between the pCT and the daily image corresponding to a later treatment fraction, regions of interest (ROI) were generated on the images such that they encompassed the same anatomical regions for each patient individually. The workflow of creating the ROIs, ensuring consistency along the images and the calculation of volumes was implemented in RayStation version 2024A‐R (RaySearch Laboratories AB, Sweden). Manipulations mainly included contouring the body (external contour), constructing ROIs, specifying points of interest (POI), and calculating rigid registrations (RR). The system's scripting capabilities allowed for automating processes, thereby ensuring consistent data collection for all patients. Once collected, the volumetric data was post‐processed and analyzed. All code required to reproduce the analysis is publicly available at https://github.com/TimFlu/ART_Volumetric/.

### 2.1 Patient database and treatment setup

The study retrospectively investigated 21 patients with H&N cancer, of whom 14 required replanning as directed by the radio‐oncologists based on mask fitting and weight loss during ongoing treatment. The treatments were performed using a Radixact (Accuray Inc., Sunnyvale, USA) equipped with ClearRT imaging system with prescribed doses ranging from 50 to 70 Gy and were divided into 17 to 33 fractions. Replanning occurred between the 9th and 27th fractions.

### 2.2 Generating ROI

To accurately evaluate changes over the course of treatment, it was essential to consistently define the same ROI individually for each patient across all daily images while ensuring that the regions contain the clinically relevant anatomy (see overview in Figure 2). This required a common reference point on all images to define the ROI center, together with consistent ROI dimensions. First, for each patient, external contours were automatically generated on the pCT and all daily images. RR focused on bone structures were then computed between each daily image and the pCT. To get the reference point on all images, we initially defined it on the pCT and subsequently propagated it to each daily image using the corresponding RR (Figure 2a). To determine the longitudinal coordinate of this reference point, we leveraged the fact that the daily images were centered on the treatment region and therefore covered a common anatomical location. We placed an initial point at the geometric center of the first daily image and mapped it to the pCT using the rigid registration. The reference point was intended to lie centrally beneath the H&N so that the resulting ROI would surely encompass this anatomy. However, the geometric center of the first daily image was not always aligned with the in‐plane center of the H&N. For this purpose, the eyes were automatically contoured on the pCT, providing a simple anatomical landmark. At this longitudinal level, a thin ROI was generated and intersected with the external contour. The geometric center of the resulting contour intersection was used as an estimate of the in‐plane center of the head, which is approximately aligned with the neck. The initial reference point retained its mapped longitudinal coordinate, while its in‐plane coordinates were replaced by this estimated head center (Figure 2a; the red reference point is aligned below the green head‐center marker). The resulting point was then used as the common center for ROI generation and was mapped from the pCT to all daily images.

**FIGURE 2:**
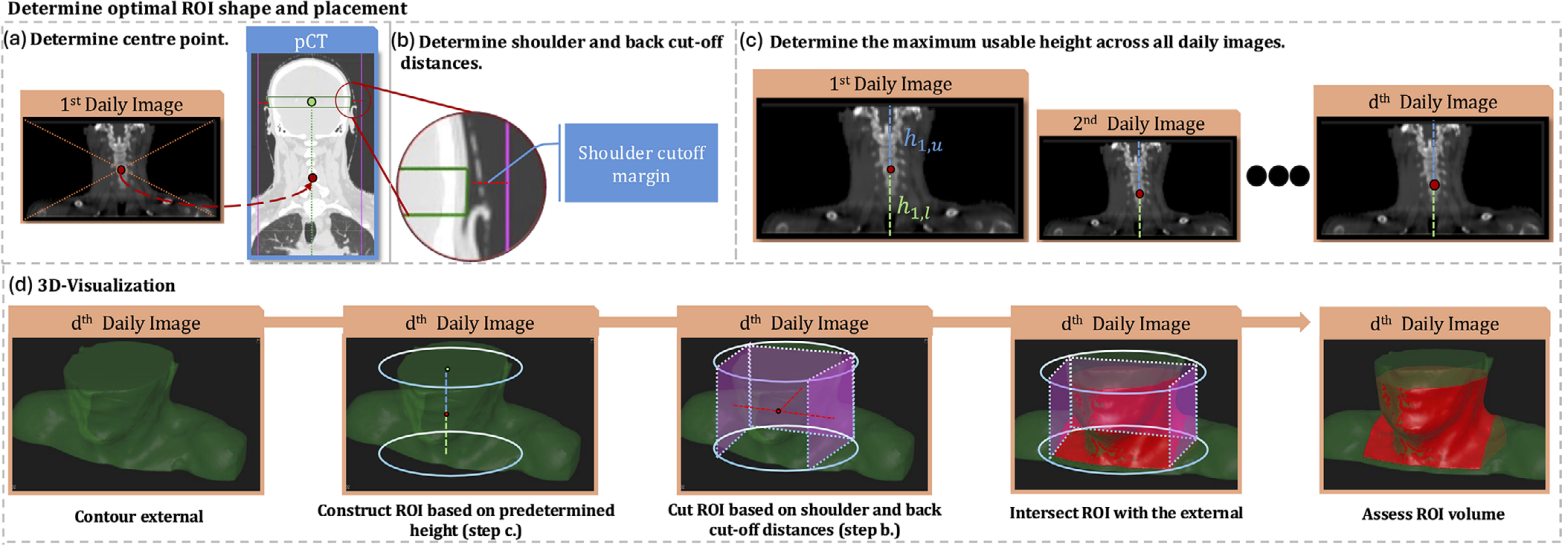
Schematic figure depicting the generation of consistent ROIs across all images for a patient. a. To define a common reference point (red dot), the centre of the first daily image is mapped to the pCT through a rigid registration and is aligned below the centre of the head (green dot). b. Due to misalignment in shoulder positioning and back‐couch intersection across images, these regions are excluded. To this end, the in‐plane head dimensions at eye level are determined and expanded by a small margin. c. The previously adjusted reference point is mapped to all remaining available daily images, again using rigid registrations. The daily images can have slightly different image modalities; therefore, the maximally possible height is defined as the smallest distance from the mapped point to either the highest or lowest slice. d. A 3D visualization of the ROI construction, starting from contouring the external, to determining the height and widths of the ROI to intersecting the generated ROI with the patients' externals, resulting in an image overarching consistent ROI covering the neck area while incorporating as much information as possible.

The ROI dimensions (width, depth, and height) were determined based on the following considerations. As shoulder positioning and back‐couch intersection can vary across images, the in‐plane dimensions were chosen such that these regions were excluded, with a small margin from the head (Figure 2b). To this end, the ROI at eye level was revisited, and the distances from the head center to the posterior and lateral boundaries of the head were determined. Margins of 1 cm posteriorly and 5 cm laterally (2.5 cm on each side) were then added to ensure full inclusion of the neck while minimizing the influence of shoulder and back positioning. The same margins were applied to all patients; while somewhat conservative for patients with lower BMI, they can be adjusted on a per‐patient basis to further reduce the influence of positional variability.

Due to slight variations in daily image coverage, the ROI height was constrained to ensure the ROI fit within the field of view across all available daily images (Figure 2c). The maximum feasible height was determined by measuring the longitudinal distances from the reference point to the lowest and highest slices of each daily image, corresponding to the lower and upper longitudinal limits of the field of view. The smallest of these distances was then selected and doubled to ensure a consistent ROI height across all images. At this stage, we had a common reference point on all images that served as the center for constructing the ROI (Figure 2d) and combined with the previously determined dimensions, this approach ensured consistent positioning and sizing of the ROIs, such that they cover the same anatomical location across all images. Finally, after intersecting the ROIs with the patient's external contours, the ROI volumes were computed for each image, and the relative volume change at fraction k with respect to the pCT was calculated as

(1) $$\Delta V_{f_{k}}=\frac{V_{f_{k}}-V_{\mathrm{pCT}}}{V_{\mathrm{pCT}}}.$$

### 2.3 Comparison of volumetric changes between replanned and non‐replanned treatment protocols

After construction of the ROIs in the initial phase, the patients were divided into two groups for an exploratory analysis: those who underwent replanning during treatment (based on radio‐oncologist's decision) and those who followed non‐replanned treatment protocols. At each treatment fraction, the relative volume changes of the patients with an available measurement were compared between groups using Welch's unequal‐variance two‐sample t‐test. Degrees of freedom were obtained via the Welch‐Satterthwaite approximation. Fraction‐wise comparisons were performed to identify when differences in mean volumetric change between the groups became apparent. Because each fraction was analyzed separately, this exploratory analysis did not explicitly model individual longitudinal trajectories or within‐patient correlations across fractions. Moreover, because a separate hypothesis test was performed for every fraction, raw p‐values were adjusted with the Benjamini‐Hochberg false‐discovery rate (FDR) procedure^14^ with q=0.05 as the significance threshold. Evidence for a difference in mean volumetric change was concluded for fractions with adjusted p‐values of $q_{FDR}\leq 0.05$.

### 2.4 Model construction and training for automated detection of relevant volumetric changes

Based on the previous evaluations, an automated workflow that triggers a warning if volumetric changes exceed norms during the treatment was implemented. To create such a workflow, a simple model was trained and evaluated on the patient database through a leave‐one‐out cross‐validation (LOOCV) scheme by optimizing a tailored loss function aimed at maximizing early detection and minimizing false negative flags.

#### 2.4.1 Data and features

The patient database was prepared by labelling patients with zero if they did not undergo replanning during their treatment, and with an integer representing the actual replan fraction if they underwent replanning. Next, the relative volumetric changes of each patient throughout the treatment were calculated as described in Section 2.2 and did serve as input for the model. To ensure maximal interpretability of the model, three features were defined based on previous knowledge about factors that necessitate replanning and are summarized in Table 1. The first feature was defined as the mean over the last two measurements, giving an estimate of the current volume loss while smoothing out outliers. The second feature corresponded to the slope of a linear regression over the last five measurements. This approach further mitigated outliers and

**TABLE 1:** Overview of the extracted features that serve as input to the flagging model, including current volume loss and short‐ and long‐term trends derived from volumetric measurements.

Features	Description
**Feature 1**: Current volume loss	Mean over the last two measurements
**Feature 2**: Short‐term trend	Slope of a linear regression fitted to the last five measurements
**Feature 3**: Long‐term trend	Slope of a linear regression fitted to all measurements

only triggered replanning if a constant trend was observed. The final feature was the slope across all measurements. This feature was particularly relevant toward the end of treatment, when the current weight loss and short‐term trends alone may not justify replanning because only a limited number of fractions remain. The resulting feature vector is summarized in the following equation:

(2) $$f\;:R^{d}\to R^{3},\;\left(\begin{array}{c} \Delta V_{f_{1}}\\ \Delta V_{f_{2}}\\ :\\ \Delta V_{f_{d}} \end{array}\right)\mapsto \left(\begin{array}{c} \frac{1}{2}\left(\Delta V_{f_{d}}+\Delta V_{f_{d-1}}\right)\\ m_{f_{d-4:d}}\\ m_{f_{1:d}} \end{array}\right),$$

where ${\left(\Delta V_{f_{1}},\Delta V_{f_{2}},\text{…},\Delta V_{f_{d}}\right)}^{T}$ is a vector containing the volume losses of the first *d* fractions relative to the pCT.

#### 2.4.2 Model

The model m was constructed to initially evaluate volumetric changes after the first five fractions of the treatment and to subsequently incorporate additional information with each new fraction. This approach ensured that the treatment flow was respected by using only data previously acquired for each treatment day. To further enhance model interpretability, the derived features (Equation (2)) were compared with corresponding thresholds.

We define a binary trigger function $g(X;k,\vec{T})$ that determines whether replanning is indicated at fraction k:

$$g\;\left(X;k,\vec{T}\right)=\left\{\begin{array}{cc} 1, & \mathrm{if}\;x_{k,1}\;<\;T_{1}\wedge x_{k,2}<T_{2}\wedge \;x_{k,3}<T_{3}\\ 0, & \mathrm{otherwise}, \end{array}\right.$$

where X is the d×3 feature matrix, with *d* denoting the maximum number of treatment fractions for that patient. The k‐th row of ***X*** contains the three feature values at fraction *k*, $x_{k,j}$ denotes the value of feature *j* at fraction *k*, and 
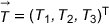
 is the vector of feature thresholds. The model output is the earliest fraction at which all three trigger conditions are satisfied:

$$m:R^{d\times 3}\to \left\{0\right\}\;\cup \;\left\{5,\;6,\text{…},d\right\},$$

(3) $$\begin{array}{cc} & m\;\left(X;\vec{T}\right)\\ & =\left\{\begin{array}{cc} \min\left\{k\in \left\{5,\text{…},d\right\}|g\left(X;k,\vec{T}\right)=1\right\}, & \mathrm{if}\;\mathrm{such}\;a\;k\;\mathrm{exists}\\ 0, & \mathrm{otherwise}. \end{array}\right.\; \end{array}$$

A reduction in volume produces a negative relative volume change, while progressive volume loss results in negative short‐ and long‐term slopes. Replanning is therefore indicated when all three feature values fall below their respective thresholds. The model m used all the data of a patient as input and returned either k for the k‐th fraction where the first replanning is estimated or zero if no replanning has been detected over the whole treatment. By defining a loss function based on the estimates and labels, the model was evaluated for different choices of  $\vec{T}$, allowing optimal thresholds to be determined.

#### 2.4.3 Tailored loss function and optimization

To identify thresholds that prioritize detection before the clinical replanning decision while strongly penalizing missed replanning events, we defined an asymmetric loss function. Correct predictions received zero loss. Early and late predictions were penalized linearly according to their distance from the clinical replanning fraction, with late predictions weighted more strongly. False‐positive and false‐negative predictions received separate fixed penalties, with false negatives penalized more strongly than false positives. For patient *i*, the loss is

(4) $$\begin{array}{cc} & l_{i}\;\left(\vec{T};p_{late},p_{fp},p_{fn}\right)\\ & =\left\{\begin{array}{cc} p_{late}\cdot \left(\hat{y\;}{\left(\vec{T}\right)}_{i}-y_{i}\right), & \mathrm{if}\;\hat{y}{\left(\vec{T}\right)}_{i}>y_{i}\\ 0.5\cdot \left(y_{i}-\hat{y}{\left(\vec{T}\right)}_{i}\right), & \mathrm{if}\;\hat{y}{\left(\vec{T}\right)}_{i}\leq y_{i}\\ p_{fp}, & \mathrm{if}\;\hat{y}{\left(\vec{T}\right)}_{i}\neq 0\;\mathrm{and}\;y_{i}=0\\ p_{fn}, & \mathrm{if}\;\hat{y}{\left(\vec{T}\right)}_{i}=0\;\mathrm{and}\;y_{i}\neq 0, \end{array}\right. \end{array}$$

where $\hat{y}\left(\vec{T}\right)$ is the predicted replanning fraction, given by the output of the model in Equation (3) and therefore the first fraction on which the features exceeded their respective thresholds. The label y is given by the fraction on which radio‐oncologists initiated replanning or zero if no replanning occurred. Furthermore, three hyper‑parameters were defined to tailor the loss function. To penalize a late trigger, the factor $p_{late}\geq 0.5$ was introduced, which imposed a penalty if the estimated replan fraction occurred later than the label. Additionally, the loss function allowed separate penalties for false positives ($p_{fp}$) and false negatives ($p_{fn}$). By selecting $p_{fp}<p_{fn}$, the model focused on minimizing missed flags as it was preferable to flag an extra patient rather than miss one who could benefit from replanning. Finally, the function to be optimized is composed of the sum over all n patients:

(5) $$\underset{\vec{T}}{argmin}L\left(\vec{T}\right)\;=\underset{\vec{T}}{argmin}\sum_{i=1}^{n}l_{i}\left(\vec{T}\right).$$

To find the thresholds that minimize the loss function in Equation (5), the *dual annealing* optimization algorithm implemented in SciPy^15^ was used.

#### 2.4.4 Model evaluation and selection

To assess the importance of the features, additional models using only subsets of the derived features were evaluated. These models, referred to as variants A‐D, were defined as follows: variant A corresponds to the previously defined model using all available features; variant B combined feature 1 with feature 2 (current volume loss and the slope over the last five measurements); variant C combined feature 1 with feature 3 (the slope over all past measurements); and variant D relied solely on feature 1 (the current volume loss). Model configurations were compared using LOOCV. The hyperparameter combinations for $p_{late},\;p_{fn},$ and $p_{fp}$ were selected from [0.5,1.0,1.5,2.0]×[225,300,450]×[150,200,300], subject to the constraint $p_{fp}<p_{fn}$. The candidate values were chosen to explore different relative penalties for delayed detection, missed replanning events, and unnecessary alerts while keeping the search space sufficiently small for the limited cohort size. The constraint $p_{fp}<p_{fn}$ encoded the clinical priority that failing to identify a patient who may benefit from replanning was considered more consequential than flagging an additional patient. The range of $p_{late}$ allowed late predictions to be penalized from equally to four times as strongly as the fixed early‐detection penalty of 0.5 per fraction. The false‐positive and false‐negative penalties were set on a larger scale than the per‐fraction timing penalties so that incorrect patient‐level decisions remained more influential than small temporal deviations from the documented replanning fraction. Because the intended clinical use prioritizes avoiding missed replanning cases, the LOOCV results of the evaluated configurations were ranked according to recall. Among configurations with identical recall, specificity was used to favor models that generated fewer unnecessary replanning alerts. If several configurations remained tied, the model with the earliest average detection time was selected. Remaining ties were resolved in favor of the simpler feature set.

For the calculation of recall and specificity, predictions made no more than five fractions after the clinically documented replanning fraction were counted as true positives. This tolerance accounts for uncertainty in the clinical reference, as the documented fraction reflects the timing of the physician's decision rather than the exact onset of clinically relevant anatomical change. Predictions occurring more than five fractions after the documented replanning fraction were considered false negatives because they were unlikely to provide timely support for treatment adaptation. Recall and specificity were calculated as

(6) $$\text{Recall}=\frac{\text{TP}}{\text{TP}+\text{FN}},\;\text{Specificity}=\frac{\text{TN}}{\text{TN}+\text{FP}}$$

To distinguish configurations with similar classification performance, the average early detection time was calculated among patients flagged before the clinically documented replanning fraction:

(7) $$\text{avg early detection}=\left\{\begin{array}{cc} \frac{1}{N_{\text{early}}}\sum_{i\in E}\left(y_{i}-{\hat{y}}_{i}\right), & N_{\text{early}}>0,\\ 0, & N_{\text{early}}=0, \end{array}\right.$$

where the set of early predictions is defined as

$$E=\left\{i|y_{i}>0,\;{\hat{y}}_{i}>0,\;{\hat{y}}_{i}<y_{i}\right\},\;N_{\text{early}}=\left|E\right|$$

Here, $y_{i}$ denotes the clinically documented replanning fraction for patient i, and ${\hat{y}}_{i}$ denotes the predicted replanning fraction given by Equation (3). The selected configuration was subsequently retrained on the complete dataset to determine the final feature thresholds.

## 3 RESULTS

### 3.1 Monitoring volume changes in daily images

After dividing the patients into two groups based on treatment replanning or not, the mean and standard deviation of the volume change were calculated across patients and per treatment fraction. The results are depicted in Figure 3. The FDR adjusted p‐values of the Welch unequal variance two sample *t*‐test of some selected fractions are shown in Table 2. The first fraction showing a significant difference between the replanned and non‐replanned groups was fraction 6 ($q_{FDR}$ = 0.011). From fractions 6 through 33, all comparisons remained significant after FDR correction except fraction 8 ($q_{FDR}$ = 0.099). Fractions 1–5 were not significant. The smallest adjusted p‐values occurred around fractions 22 and 25 ($q_{FDR}$ ≈ 4.3×10^−^^5^).

**FIGURE 3:**
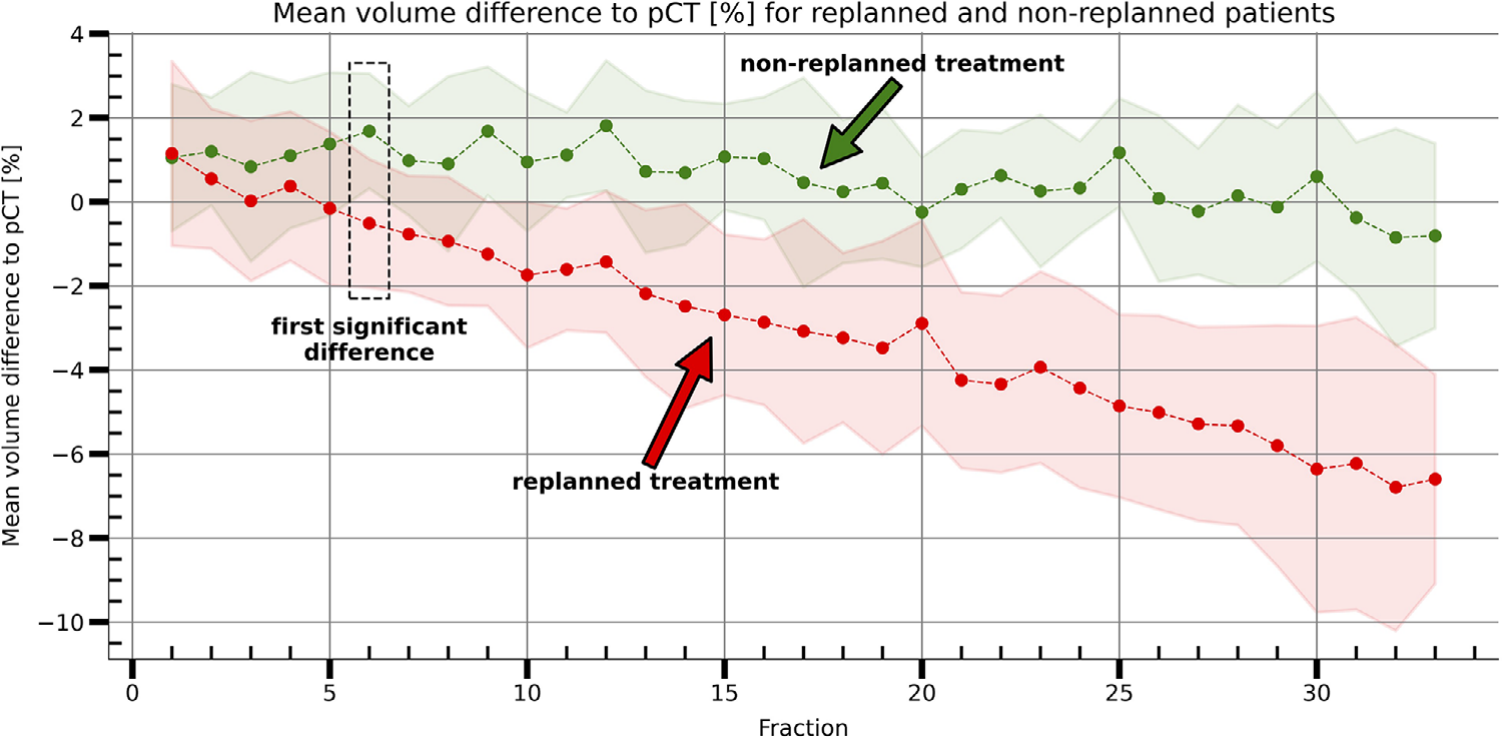
Mean relative volumetric change with respect to the planning CT for replanned (red) and non‐replanned (green) patients over the course of treatment. Shaded regions represent one standard deviation across patients at each treatment fraction, illustrating the between‐patient variability in relative volumetric change. The first statistically significant difference between the two groups was observed at fraction 6 (Welch's unequal‐variance two‐sample *t*‐test with BenjaminiHochberg correction; $q_{FDR}<0.05$) and is indicated by the dashed box.

**TABLE 2:** A selection of resulting p‐values from Welch unequal variance two sample *t*‐test with corresponding False Discovery Rate (FDR) correction. Bold indicates $q_{FDR}<0.05$ and is considered significant.

Fraction	$p_{\mathrm{raw}}$	$q_{\mathrm{FDR}}$
1	0.92	0.92
5	0.099	0.113
6	$8.0\cdot 10^{-3}$	$1.1\cdot 10^{-2}$
8	0.084	0.099
22	$3.0\cdot 10^{-6}$	$4.3\cdot 10^{-5}$
25	$2.0\cdot 10^{-6}$	$4.3\cdot 10^{-5}$
33	$7.4\cdot 10^{-4}$	$1.7\cdot 10^{-3}$

### 3.2 Model performance and selection

Five feature‐map and loss‐hyperparameter combinations with the highest LOOCV recall, specificity, and early‐flagging performance are shown in Table 3. All of them result in the same confusion matrix, indicating that the corresponding feature‐map loss‐hyperparameter combinations led to essentially identical predictive behavior. The primary difference between the variants lies in how early they detect the need for replanning. The variant which used all three features while imposing a four‐fold stronger penalty on late flagging than on early flagging, assigning a penalty of 450 for flagging more than five fractions after the physician initiated replanning or for missing the event entirely and while using a three‐times lower factor for false positives outperformed the others slightly. After retraining this hyperparameter variant on the full dataset, the final model thresholds are $\vec{T}={\left(-2.11\%,\;-0.45\frac{\%}{\mathrm{frac}},\;-0.1\frac{\%}{\mathrm{frac}}\right)}^{T}$.

**TABLE 3:** The five highest‐ranked models based on LOOCV recall, followed by specificity and early flagging. For each feature‐set and loss‐hyperparameter combination, the individual performance metrics are reported. The asterisk marks the configuration selected for subsequent training.

variant	$p_{\mathrm{late}}$	$p_{\mathrm{fn}}$	$p_{\mathrm{fp}}$	recall	specificity	avg early detection
A*	2.0	450	150	0.93	0.86	8.4
A	2.0	300	150	0.93	0.86	8.3
A	1.5	450	150	0.93	0.86	8.3
A	1.5	300	200	0.93	0.86	8.2
B	0.5	300	150	0.93	0.86	7.4

## 4 DISCUSSION

This study proposes a lightweight and interpretable method to help radio‐oncologists argue for the safe continuation of a patient's treatment. By automatically generating ROIs around the target on daily images and the pCT, critical soft‐tissue changes can be detected using a simple model that computes interpretable features and compares them to predefined thresholds. These features capture clinically relevant soft tissue changes without requiring deformable registrations, synthetic CT generation, or treatment plan re‐calculations. Moreover, the workflow requires only limited manual validation, primarily visual verification of the automatically generated external contours. Potential failure modes arise from insufficient contour quality or from shoulder and posterior cut‐off margins that do not adequately exclude regions affected by positional variability. In this study, conservative fixed margins were applied to all patients to reduce the influence of shoulder position and couch intersection. In special cases, these margins could be adjusted on a patient‐specific basis.

A statistically significant difference in volume changes between replanned and non‐replanned patients was observed across daily images as early as the sixth treatment fraction, supporting the hypothesis that early anatomical deviations are predictive of later physician‐initiated replanning. Under LOOCV, the best model flagged 93% of patients who were eventually replanned, and flagged 77% of them early, on average 8.4 fractions before the clinical intervention. After retraining on the full dataset, the final trigger model recommended replanning when (i) the average volumetric change over the two most recent measurements fell below –2.11%, (ii) the short‐term trend over the previous five fractions exceeded a loss of 0.45% per fraction, and (iii) the longer‐term trend exceeded 0.1% per fraction. These interpretable criteria reflect progressive tissue loss, offering transparency that is rarely achieved with more complex approaches.

In current clinical practice, replanning decisions are often based on qualitative indicators, such as visible weight loss or changes in mask fit. However, there is no standardized quantitative threshold for determining when replanning should be performed, and this reliance on subjective assessment leads to variations from clinician to clinician and from center to center, underscoring the need for objective, imaging‐based indicators.

Several imaging‐based approaches to flag replanning necessity during radiotherapy have been proposed to address this gap. The most direct strategy, acquiring scheduled simulation CT scans during treatment to monitor anatomical changes and recalculate dose,^16^ would provide the highest‐quality information but is impractical in clinical practice due to cost, workflow burden, and the additional ionizing radiation delivered to the patient.^17^ Methods based on synthetic CT generation enable contour propagation and dose recalculation, but they typically rely on deep‐learning models that are difficult to interpret and often sensitive to variations in scanner type, acquisition protocol, and to the propagation of artifacts inherent to daily imaging.^18^

Other approaches combine rigid or deformable image registration with dose recalculation, mapping the pCT, its contours, or its dose distribution onto daily images and re‐estimating the dose to quantify changes in target coverage and organ‐at‐risk exposure.^8^, ^19^, ^20^, ^21^, ^22^, ^23^ While DIR based workflows can capture deteriorating dose coverage, their reliability is fundamentally constrained by registration uncertainty. Inaccurate deformation fields may arise from daily image artifacts, poor soft‐tissue contrast, or anatomical changes that violate the assumptions of the registration model. These uncertainties propagate directly into estimated target coverage and accumulated dose, and the absence of a ground‐truth deformation field makes it impossible to fully quantify their accuracy.^24^ As a result, DIR‐based dose tracking remains sensitive to imaging quality, the choice of registration algorithm and requires a rather complex quality assurance.

Considering these limitations, the simple volumetric trigger presented in this work offers a complementary and transparent alternative. The method focuses on robust geometric descriptors that can be computed consistently on daily imaging. The algorithm is not intended to replace clinical judgement but to support it through flagging anatomically meaningful deviations early in the treatment course.

This proof‐of‐concept study nevertheless has several limitations. First, the model was developed in a small retrospective cohort of 21 patients, including only seven patients who did not undergo replanning. Model configurations were compared using LOOCV. Although appropriate for exploratory evaluation in this small cohort, this internal validation does not replace assessment on an independent external dataset. The selected model was subsequently retrained on the complete cohort, and its final thresholds were therefore not evaluated on an independent test set. Furthermore, all data originated from a single institution using one treatment and imaging system, and no external cohort was available to assess generalizability across institutions, imaging protocols, treatment techniques, or patient populations. Second, physician‐initiated replanning was used as the reference standard. This endpoint reflects clinical decision‐making in routine practice but does not necessarily correspond to the exact onset of clinically relevant anatomical or dosimetric deterioration. Decisions may be influenced by subjective assessment, mask fit, weight loss, workflow constraints, and clinician‐specific practice. Consequently, the reference labels may contain temporal uncertainty and may differ from the underlying need for adaptation. Third, the method depends on the quality and consistency of the automatically generated external contours. It also assumes that the pCT includes the eyes, which are used as an anatomical landmark for defining the in‐plane head center, and that the daily images provide sufficient and broadly consistent coverage of the relevant head‐and‐neck region. If these conditions are not met, the reference‐point definition and ROI construction may fail or become less reliable. The method captures external volumetric changes but may not detect internal anatomical changes that do not substantially alter the external contour. Future studies should therefore validate the method in larger, independent, and more diverse cohorts and compare its predictions against dosimetric endpoints, such as changes in target coverage or organ‐at‐risk dose.

## 5 CONCLUSIONS

This study demonstrates that soft‐tissue changes within the head‐and‐neck region, extracted from daily imaging, provide a reliable and interpretable basis for identifying patients who may benefit from replanning during radiotherapy. The proposed method captures simple volumetric based features, offering an objective trigger that complements clinician judgement while avoiding the uncertainties and computational demands of synthetic CT generation, deformable image registration, and repeated dose recalculation. By operating directly on images already acquired in standard workflows, the approach represents a practical, easy to implement and transparent tool for monitoring anatomical evolution throughout treatment.

Further validation in larger and more diverse patient cohorts will be essential to assess the generalizability of the results and determine whether the algorithm's predictions, based on anatomical changes, can translate into clinically significant improvements in terms of target coverage, preservation of at‐risk organs, and overall adaptive treatment strategies.

## CONFLICT OF INTEREST STATEMENT

Stina Svensson and Anna Lundin are employees of RaySearchLabs. RaySearchLabs partially funded that study.
